# Operational Tree Species Mapping in a Diverse Tropical Forest with Airborne Imaging Spectroscopy

**DOI:** 10.1371/journal.pone.0118403

**Published:** 2015-07-08

**Authors:** Claire A. Baldeck, Gregory P. Asner, Robin E. Martin, Christopher B. Anderson, David E. Knapp, James R. Kellner, S. Joseph Wright

**Affiliations:** 1 Department of Global Ecology, Carnegie Institution for Science, Stanford, CA, United States of America; 2 Department of Ecology and Evolutionary Biology, Brown University, Providence, RI, United States of America; 3 Smithsonian Tropical Research Institute, Washington, DC, United States of America; University of New England, AUSTRALIA

## Abstract

Remote identification and mapping of canopy tree species can contribute valuable information towards our understanding of ecosystem biodiversity and function over large spatial scales. However, the extreme challenges posed by highly diverse, closed-canopy tropical forests have prevented automated remote species mapping of non-flowering tree crowns in these ecosystems. We set out to identify individuals of three focal canopy tree species amongst a diverse background of tree and liana species on Barro Colorado Island, Panama, using airborne imaging spectroscopy data. First, we compared two leading single-class classification methods—binary support vector machine (SVM) and biased SVM—for their performance in identifying pixels of a single focal species. From this comparison we determined that biased SVM was more precise and created a multi-species classification model by combining the three biased SVM models. This model was applied to the imagery to identify pixels belonging to the three focal species and the prediction results were then processed to create a map of focal species crown objects. Crown-level cross-validation of the training data indicated that the multi-species classification model had pixel-level producer’s accuracies of 94–97% for the three focal species, and field validation of the predicted crown objects indicated that these had user’s accuracies of 94–100%. Our results demonstrate the ability of high spatial and spectral resolution remote sensing to accurately detect non-flowering crowns of focal species within a diverse tropical forest. We attribute the success of our model to recent classification and mapping techniques adapted to species detection in diverse closed-canopy forests, which can pave the way for remote species mapping in a wider variety of ecosystems.

## Introduction

A comprehensive understanding of tropical forest biodiversity and function over large spatial scales is hindered by a lack of spatially extensive information on tree species composition, as field measurements are often spatially localized and have difficulty resolving large geographic patterns [1]. Remote sensing can extend field-based knowledge over large areas by providing continuous spatial coverage of surface reflectance and structural properties [2,3]. In particular, imaging spectroscopy (or hyperspectral remote sensing) measures light reflected in hundreds of narrow, contiguous spectral bands, which may allow the discrimination of different plant species. However, the use of imaging spectroscopy for remote species mapping presents a major practical challenge in tropical forests where the species richness of trees ≥ 10 cm dbh may exceed 300 species per ha [4]. As automated tree species mapping has not yet been accomplished in a high diversity tropical forest, we currently have little understanding of the ability of imaging spectroscopy to map species in these ecosystems.

To assess the potential for remote species mapping in tropical forests, a number of studies have examined the spectral separability and classification performance of different tropical plant species and growth forms. Spectral differences have been found among leaf- and branch-level spectra of different species from the same ecosystem, as well as the spectra of different species detected from hyperspectral airborne and satellite sensors [5–11]. Important spectral differences have also been found between tree and liana species [12,13], as well as between groups of native and invasive species [14]. These results indicate the potential for classification models to differentiate species or groups of species based on their remotely-sensed spectral signatures.

In a foundational study, Clark et al. [7] attempted to classify seven canopy tree species from a diverse Costa Rican rainforest based on their remotely-sensed hyperspectral signatures collected with the airborne sensor HYDICE. Their most successful model classified the crowns of their study species with 92% accuracy. Despite these encouraging results, this classification model could not be used to map any of the seven study species across the study site. The primary reason for this was that the classification model differentiated the seven study species from one another, but not from the hundreds of other tree and liana species present at the site. In addition, the preferred model was based on crown-mean spectra, and thus operational species mapping would require automatic delineation of tree crowns throughout the image prior to applying the classification model to predict species identity. At the time of the study, the ability to accurately, automatically delineate individual tree crowns in a closed-canopy tropical forest did not exist, and nearly a decade later it still does not.

Since Clark et al. [7], others have performed successful classifications of tropical forest species based on their remotely-sensed spectral signatures, but these models were also non-operational in that they were unable to distinguish the study species from the other species present at the study site, e.g., [15]. Meanwhile, operational species mapping has been accomplished in several ecosystems with relatively low species diversity [16–20]. The inability of operational species mapping to move beyond lower diversity ecosystems may lie in the classification approach used. In the aforementioned classification and mapping studies, species classification models were based upon the traditional multi-class classification approach, which requires representative training data to be collected for all model classes (species) present in the scene. This requirement is not likely to be fulfilled in tropical forests that harbor hundreds to thousands of species. Even if representative training data were available for all canopy species in a high-diversity forest, the outlook for such a classification model would be quite dim as classification accuracy is known to decline as the number of classes increases [18,21]. In the face of these mounting challenges, it is not surprising that the remote sensing community has expressed ambivalence towards the prospect of remote species mapping in high diversity tropical forests [22].

Clearly, a different approach is needed to accomplish operational remote species mapping in high-diversity forests. A possible solution may come from single-class classification methods that distinguish samples of a single focal class from those of all other classes, e.g., [23–25]. These methods are advantageous when the interest is in mapping a single class because they aim to maximize classification accuracy with respect to the focal class rather than the average classification accuracy over all model classes. They also reduce the overall amount of training data required compared to traditional multi-class classification, and some require training data from only the class of interest [23,24], making acquisition of an appropriate training dataset more feasible for tropical forests. Moreover, single-species classification models can be directly applied for species mapping in the area of study because they explicitly aim to distinguish the focal species from all other species. Thus far, single-class classification methods have been explored for species classification and mapping with promising results in a few ecosystems with relatively low species diversity [25–27]. If these methods are found to be effective in high-diversity ecosystems, single-species classification may solve many of the problems associated with remote species mapping in tropical forests.

Taking a single-species detection approach, we set out to map three important canopy tree species within the diverse, closed-canopy tropical forest of Barro Colorado Island, Panama, using high spatial resolution imaging spectrometer data. We evaluated two leading single-class classification methods (discussed in detail in Methods section) for their ability to distinguish the remotely-sensed spectra of the focal species from a diverse background of other tree and liana species based solely on their non-flowering crown spectral characteristics. We combined the selected single-species models into a multi-species model for mapping the three focal species across the island and evaluated the predictions by confirming the species identities in the field. Our species maps were highly reliable, demonstrating that tropical tree species can be effectively mapped with airborne imaging spectroscopy data. Together, the techniques presented here represent a high-performance, operational method for the identification of individual tree crown objects of one or more focal species that is adapted to the challenges encountered in tropical forests and greatly reduces the burden of training data collection.

## Methods

### Study site

**Fig 1:**
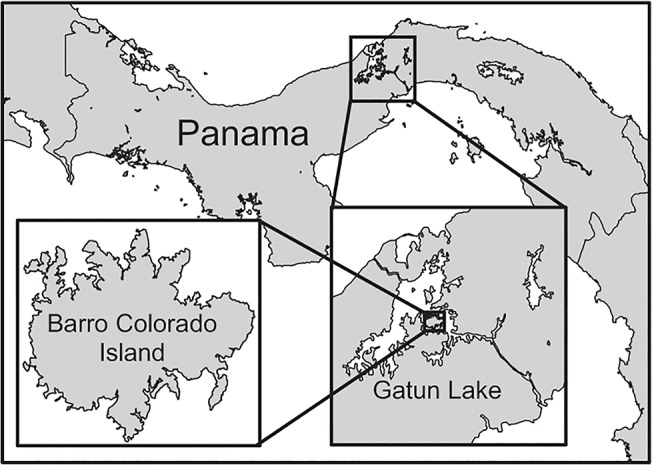
Map of Barro Colorado Island (BCI), Panama.

Barro Colorado Island (BCI) is a 1560-ha island located in Gatun Lake, which was formed in the early 20^th^ century as part of the construction of the Panama Canal (Fig 1). The BCI forest is tropical moist, with a mean annual temperature of 26°C and mean annual rainfall of 2600 mm. There is a distinct dry season from January to April, and an estimated 6.3% of trees > 30 cm dbh are deciduous during the dry season [28]. There are approximately 500 tree species in the BCI forest [29,30], with nearly 60 tree species ha^-1^ among trees ≥ 20 cm dbh [31], a size typically considered to occupy the canopy. In addition to the high species diversity of trees present in the canopy, there are many lianas present in this forest. Less is known about the diversity of lianas at the top of the canopy, but a census of liana stems on BCI found an average of 75 liana species ha^-1^ for stems ≥ 1 cm in diameter [32]. Surveys of liana infestation of trees have estimated that ~78% of trees ≥ 20 cm dbh have at least one liana in their crown, though not necessarily overtopping the crown [33].

### Spectral data

Imaging spectrometer data were collected over Barro Colorado Island, Panama, with the Carnegie Airborne Observatory Airborne Taxonomic Mapping System (CAO AToMS; [34]) in February of 2012. The CAO was flown at an altitude of 1 km providing data at 1.12 m spatial resolution, and was flown between 10 am and 1:30 pm under nearly cloud-free conditions to minimize shadow effects. The CAO AToMS system consists of three instrument subunits: i) a High-fidelity Imaging Spectrometer (HiFIS), ii) a Light Detection and Ranging (LiDAR) scanner, and iii) a Global Positioning System-Inertial Measurement Unit (GPS-IMU). The CAO HiFIS subsystem provided spectroscopic images spanning the visible to shortwave infrared portion of the spectrum between 380 and 2512 nm, which was resampled to 10.4 nm spectral resolution. The LiDAR subsystem was operated in discrete return mode with a density of four laser shots per square meter with up to four returns per shot, providing three-dimensional information on terrain and canopy structure [34]. The GPS-IMU subsystem provided positioning and attitude data to project the HiFIS and LiDAR data onto the land surface. Canopy height information provided by the LiDAR subsystem was used for accurate orthorectification of the spectral data. Radiance data were converted to surface reflectance using ACORN 5BatchLi (Imspec LLC, Palmdale, CA) with a MODTRAN look-up table and reflectance was BRDF adjusted to correct for cross-track reflectance gradients [35]. Prior to analysis, we removed spectral bands that were heavily influenced by water absorption (1330–1460 nm and 1770–2022 nm) and noisy bands at the upper and lower portions of the spectrum (below 400 nm and above 2422 nm). This resulted in a total of 167 spectral bands used for analysis.

A field campaign was conducted to locate and identify individual tree crowns within the CAO image in January of 2013. Crown locations were recorded in the field with a handheld Global Positioning System (GS50 Leica Geosystems Inc., Norcross, GA, USA). These crowns were then manually delineated in the CAO imagery, taking care to ensure that the pixels extracted were as pure as possible, without contamination from lianas or neighboring trees. The crowns were collected along trails traversing the full east-west span of the island, sampling across all flight lines. The campaign was conducted to sample the tree diversity of the island as widely as possible, without sampling large numbers of any one species. This resulted in 340 crowns representing 84 tree species. Although not used in this study, leaves were collected from identified crowns with a permit from the Smithsonian Tropical Research Institution to Robin Martin.

To obtain a larger sample of crowns for a few key species, we used satellite imagery collected over the island from the Ikonos, QuickBird, and WorldView-2 sensors. Three common canopy tree species–*Dipteryx panamensis*, *Handroanthus guayacan* (formerly assigned to the genus *Tabebuia*), and *Jacaranda copaia*–flower at different times of the year. *D*. *panamensis* flowers in June-August, *H*. *guayacan* flowers after heavy rainfall events in the second half of the dry season, and *J*. *copaia* flowers in March-April before the onset of the wet season. In these events, many mature canopy individuals will display colorful flowers that allow them to be identified in multispectral satellite images or aerial photographs. Individuals of these species were identified in a series of satellite images acquired between 2002 and 2011 during flowering events [36]. Locations of crowns identified from the satellite images were superimposed on the CAO image and 40 crowns of each species were manually delineated in the CAO image. Crowns transferred to the CAO image were distributed evenly across the island while avoiding proximity to trails (< 20 m). Crowns of *H*. *guayacan* and *J*. *copaia* were checked in the field at the time of the CAO flight and both species were found to have foliage but not flowers. Thus our efforts to identify these species using imaging spectroscopy are based solely on the optical reflectance properties of non-flowering tree crowns.

**Fig 2:**
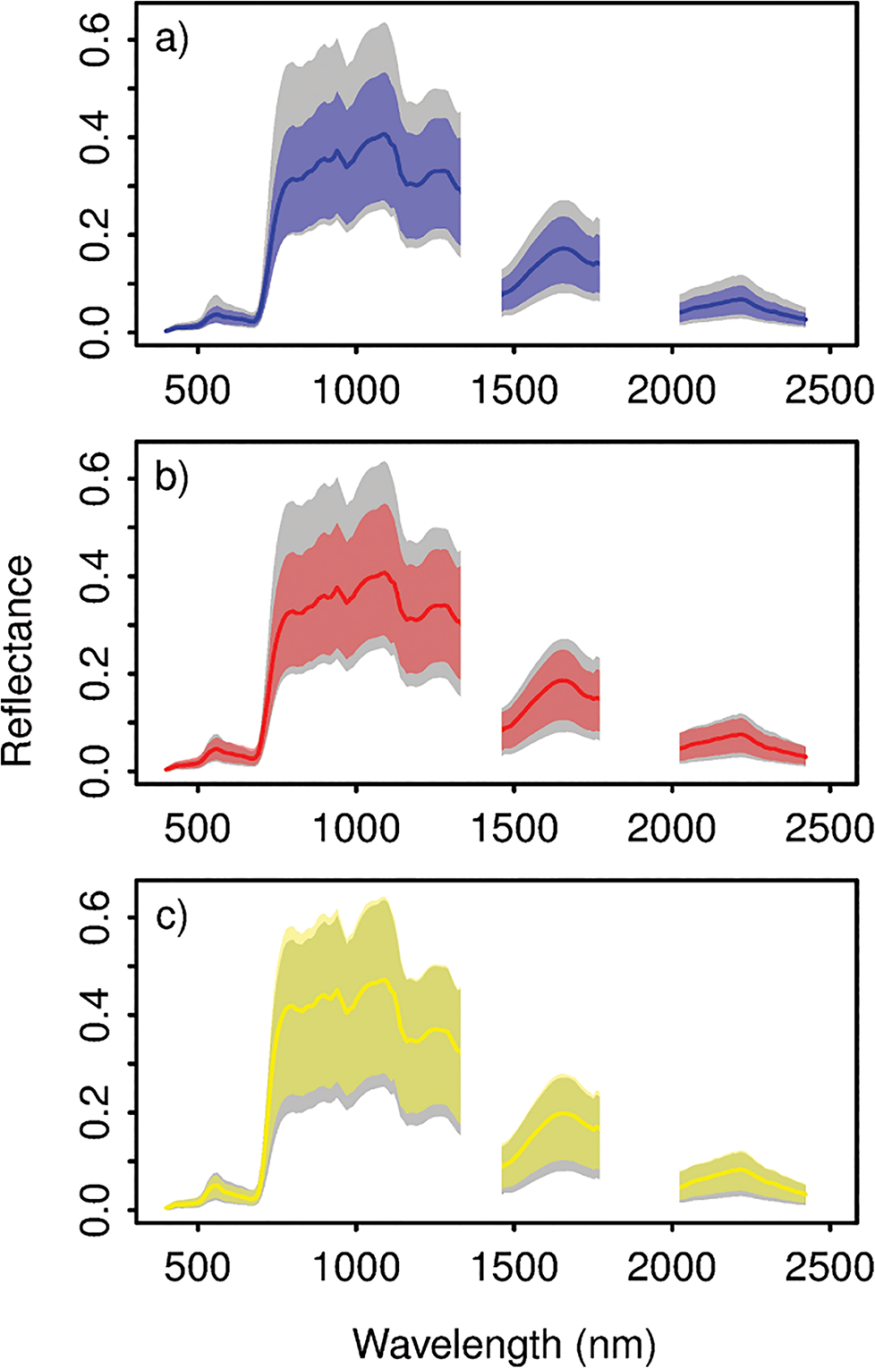
Mean and 95% confidence interval of the reflectance spectra for each focal species. (a) *Dipteryx panamensis*, (b) *Handroanthus guayacan*, and (c) *Jacaranda copaia*. The colored lines represent the mean reflectance, and the colored areas represent the 95% confidence interval for each spectral band. The gray area represents the 95% confidence interval for the well-lit leafy vegetation on BCI.

The field-collected crowns and those obtained from the satellite data were combined into a single dataset to compare methods for single species identification and to map the focal species across BCI. Each of the three focal species was well represented by over 40 training crowns, while the field-collected crowns provided a broad sampling of the tree species across the island. Prior to analysis, all of the spectral data were filtered to include only well-lit, leafy vegetation pixels with NDVI ≥ 0.7 and mean NIR (850–1050 nm) reflectance ≥ 21% (referred to here as “vegetation pixels”). A summary of the spectral data is presented in Table 1 and the mean and 95% confidence interval of the spectra for the three focal species is shown in Fig 2, superimposed on the 95% confidence interval for the spectra of all vegetation pixels on the island.

**Table 1:** Summary of the spectral data from both the field-collection campaign and the crowns obtained from satellite data.

Species	# Crowns	# Pixels
*D*. *panamensis*	50	10726
*H*. *guayacan*	44	3325
*J*. *copaia*	44	2091
Other	322	9350
Total	460	25492

### Single-species classification methods

We investigated two support vector machine (SVM) techniques that have shown promise in distinguishing samples of a single focal species from those of all other species–binary SVM and biased SVM [26]. The basic SVM is a non-parametric classification method that is widely used in remote sensing because of its good classification performance and elegant handling of high-dimensional data [37–39]. It has also been shown to be among the top-performing classification methods in remote species identification [17,18,40]. SVM is a binary classifier that separates samples of two classes in feature space with a decision boundary [41,42]. This boundary is generated by maximizing the margins between the boundary and the closest training samples (the support vectors), while minimizing misclassification errors. A fitted cost parameter, *C*, controls the penalty associated with misclassification error and thus the complexity of the decision boundary; a higher misclassification penalty will result in a more complex model, but with more danger of overfitting. A kernel function may be used to perform an implicit mapping of the data into a higher dimensional feature space [43,44], allowing non-linear separation of the classes in the original feature space. The popular radial basis function (RBF) kernel has a single fitted parameter, γ, controlling the kernel width. Thus, constructing an SVM with an RBF kernel requires fitting of two parameters, γ and *C*. Optimal values of these parameters are found through cross-validation on the training dataset.

For this analysis we used a specific type of binary SVM in which samples from a single class of interest, or the focal class, are contrasted against samples from all other classes, which together make up the non-focal, or outlier, class. This is also referred to as “one-against-all” binary SVM. The F-score on the focal class (F = 2*pr*/(*p*+*r*), where *r* = sensitivity or recall accuracy and *p* = precision) is an appropriate choice of optimization criterion in single-class classification with highly imbalanced class sizes. Sensitivity is the proportion of focal class samples that are correctly assigned by the classifier to the focal class (i.e., the producer’s accuracy for the focal class) and precision is the proportion of samples assigned to the focal class that truly belong to the focal class (i.e., the user’s accuracy for the focal class). Maximization of the F-score selects a combination of parameter values that yields both high sensitivity and high precision, while keeping these two aspects of model performance relatively well balanced.

Biased SVM [24] works similarly to the standard binary SVM in that it finds an optimal separation between two classes in feature space. However, the collected training data from the focal class are contrasted against samples that are randomly selected from the data pool (in this case, the vegetation pixels from the entire island), referred to here as “pseudo-outliers”. Because the pseudo-outlier data are of unknown identity and will contain samples from the focal class, errors occurring within the pseudo-outlier class are given a lower penalty than errors occurring within the focal class. An extra parameter, *w*_*c*_, is added to the model, which controls the relative cost of errors occurring in the two classes.

The parameterization of biased SVM is also performed through cross-validation on the training dataset. However, without known samples from the outlier class, precision cannot be calculated. A tuning criterion that works well for biased SVM is *r* ^2^/P[ƒ(x) = 1], where *r* is the sensitivity (recall accuracy) and P[ƒ(x) = 1] is the probability that a sample is assigned to the focal class [26,45]. This criterion is based on the concept that model sensitivity should be maximized while simultaneously minimizing the number of samples that are assigned to the focal class, and is thought to work similarly to the F-score in that it will be large when both recall and precision are large [45]. In a study examining the optimization of biased SVM for remote tree species mapping, Baldeck and Asner [26] found that this criterion worked well for optimizing biased SVMs and generally outperformed an alternative optimization criterion. They also found that cross-validation generally worked best when performed at the crown level (i.e., by splitting crowns rather than pixels into the cross-validation groups).

One-against-all binary SVM and biased SVM have thus far been applied to species detection in only a few instances [26,27]. Binary SVM was initially used in a study exploring different methods for species detection in a Hawaiian forest [27]. Biased SVM is a relatively new single-class classification method that first showed promise in studies of web document and land-cover classification [24,46], and represented an attractive possibility for species detection because it could be trained with a fraction of the training data used in binary SVM. Only one study has investigated the potential of biased SVM for use in single-species detection [26]. That study compared biased SVM to binary SVM using crown spectral data from a savanna ecosystem and found that biased SVM performed well, though it did not achieve as high overall performance as binary SVM.

### Comparison of binary versus biased SVM

To create the binary SVM models, the training data were divided into the focal species class and the non-focal, or outlier, species class. The training dataset for each focal species consisted of all the focal species crowns plus all outlier species crowns from the field collection campaign. Only crowns from the field collection campaign were used to form the outlier class to prevent skewing this class heavily toward the other focal species. It has been shown that the balance in the amount of data from the focal and outlier classes can affect model performance in this type of one-against-all binary classification [26]; therefore, we conducted a preliminary analysis to assess model balance for these datasets, which is described in the Supporting Information. We found that the ratio of focal to outlier species crowns present in these datasets (approximately 0.15) yielded good results (S1 Fig), and so the full datasets were used in the tests of the binary SVM models.

For each focal species binary SVM model, the optimal values of γ and *C* were found by an exhaustive grid search over the values γ ∈ {*e* ^-12^, *e* ^-11^, …, *e* ^-6^} and *C* ∈ {*e* ^5^, *e* ^6^, …, *e* ^15^} using five-fold crown-level cross-validation [21]. In this procedure, the crowns of each class were split into five roughly equally sized groups. One group from each class was set aside to form the test dataset and a model was built from the remaining data using the trial parameter values. This was repeated five times such that the data from each group was predicted with a model built from a separate set of crowns. The F-score on the focal class was calculated based on the number of pixels that were correctly classified and this was repeated for every parameter combination. The combination of γ and *C* resulting in the highest cross-validation F-score was chosen for the final model for each species.

To create the biased SVM models, the focal species crowns were used to form the focal class and 40,000 vegetation pixels were randomly sampled from the CAO imagery to form the pseudo-outlier class. The biased SVM models were optimized using the same crown-level cross-validation procedure described for binary SVM, except that the pseudo-outlier data were split into five groups randomly by pixel. The optimal values of γ, *C*, and *w*_*c*_ were found through a grid search over the values γ ∈ {*e* ^-8^, *e* ^-7^, …, *e* ^-2^}, *C* ∈ {*e* ^5^, *e* ^6^, …, *e* ^11^}, and *w*_*c*_ ∈ {0.1, 0.2, …, 0.7}. To save computation time, a coarse search was conducted over these ranges using every other value of each parameter, then a fine-scale search was conducted bracketing the optimal values identified from the coarse search. The biased SVMs were optimized using the *r* ^2^/P[ƒ(x) = 1] criterion estimated using all focal species and pseudo-outlier pixels [45].

Once the optimal parameters were identified, we compared the performance of the binary and biased SVM models. For binary SVM, one-fifth of both the focal and outlier crowns were removed to form the test dataset and a model was built using the remaining data using the optimal parameter values. For biased SVM, one-fifth of the focal species crowns were removed and placed in the test dataset and a model was built using the remaining focal species crowns and 32,000 pseudo-outlier pixels. Focal and outlier crowns were randomly assigned to the training and test groups and the same group assignments were used for both the binary and biased models of the same species. This procedure was repeated 100 times. We were especially interested in comparing the sensitivity and specificity (the proportion of outlier class samples that are correctly assigned to the outlier class) of the two classifiers. Therefore, although the outlier crowns were not used to train the biased SVMs, the test outlier crowns were passed to the biased SVM models to estimate specificity. We also compared the performance of binary and biased SVM by using each to map the focal species across the island. For each focal species and classifier type, a classification model was built from the full dataset and this was applied to all vegetation pixels in the CAO imagery.

### Focal species mapping

Based on our comparison of binary and biased SVM methods for mapping the focal species (see **Results**section below), we chose to use the biased SVM method as the basis for creating the final maps of the three focal species. A final biased SVM model was created for each focal species using all of the data available for that species plus all of the pseudo-outlier data. Then the three final biased SVMs were combined into a multi-species classification to be used for mapping the three species simultaneously.

As the three biased SVM models are independent of one another, it is possible for a pixel to be assigned to the focal species of more than one model. To break such ties we trained an ordinary multi-class SVM with an RBF kernel to differentiate the three focal classes from one another. All crowns from the three focal species were used, but the data from *D*. *panamensis* and *H*. *guayacan* were randomly subset to 2091 pixels (the number available for *J*. *copaia*) to create evenly balanced species classes. The biased SVMs for the three focal species and the tie-breaking SVM were combined into the final, multi-species classification model with the following rules: (i) If a pixel was assigned to the outlier class by all of the biased SVM models, then that pixel was assigned to the outlier class. (ii) If a pixel was assigned to the focal species class by only one biased SVM model, then it was assigned to the corresponding focal species. (iii) If a pixel was assigned to the focal species class by more than one biased SVM model, the pixel was passed to the tie-breaking SVM to determine the species assignment. For the final multi-species classification model, we obtained estimates of the pixel-level producer’s accuracies for the three focal species (equivalent to the sensitivity, but for multi-class models) using five-fold crown-level cross-validation. We then applied this model to all vegetation pixels in the CAO imagery to map the three focal species.

The raster of the model prediction results contained some amount of undesirable noise (spatially random pixels assigned to the focal classes). To reduce this noise, we applied contextual filters to the model prediction results. First, we resized the raster grid by cutting each pixel into quarters and performed opening-closing on the raster of predicted occurrences for each species (3 × 3 pixel kernel; ENVI software, Exelis, Boulder, CO USA). The opening procedure removed small objects of two pixels in width and then the closing procedure filled small holes of two pixels in width (here, two pixels in width is equivalent to one pixel in width at the original resolution). The raster grid was then resized to the original resolution and each contiguous area belonging to the same focal class was designated as an individual object (contiguity was defined as pixels that were side-adjacent). Objects with fewer than ten pixels were eliminated and objects that remained were counted as crown objects. We refer to these areas as “crown objects” rather than “crowns” to acknowledge that a single crown object may contain the crowns of two or more individual conspecific trees, and that the crown of one individual tree may be represented by two or more smaller crown objects, separated perhaps by liana or shadow.

### Field validation

To obtain estimates of model precision for predicted crown objects, we returned to the island to verify their species identity. The prediction map was brought to the field on a tablet PC and predicted crown objects located near trails (< 20 m away) were checked to verify their species identity. Only trail segments that were not visited in the first field campaign were used for model verification. No permits were needed to check the identities of the tree crowns on the island.

Additional field observations were recorded to help with the interpretation of the prediction map. We noted the presence of individuals of the focal species with crowns reaching the top of the canopy (at least 5 m in diameter) that were encountered in the field but not indicated by the prediction map, which might be considered as a failure of the model to detect the presence of that species. For all focal species trees encountered, the presence and severity of liana infestation of the tree crown was recorded, with special attention to whether the lianas were covering the top of the canopy. However, the field search for omitted focal species crowns was not systematic and there was ambiguity in which ones should be considered to be a genuine omission error (for example, the presence of lianas may indicate that the omission is correct from a remote sensing perspective). Therefore, we could not quantify the proportion of focal species canopy individuals that were not detected (i.e., we could not measure crown-object-level sensitivity). These observations were meant only to explore sources of error; we relied on the previously described accuracy analyses to estimate model sensitivity.

## Results

### Comparison of binary versus biased SVM

**Fig 3:**
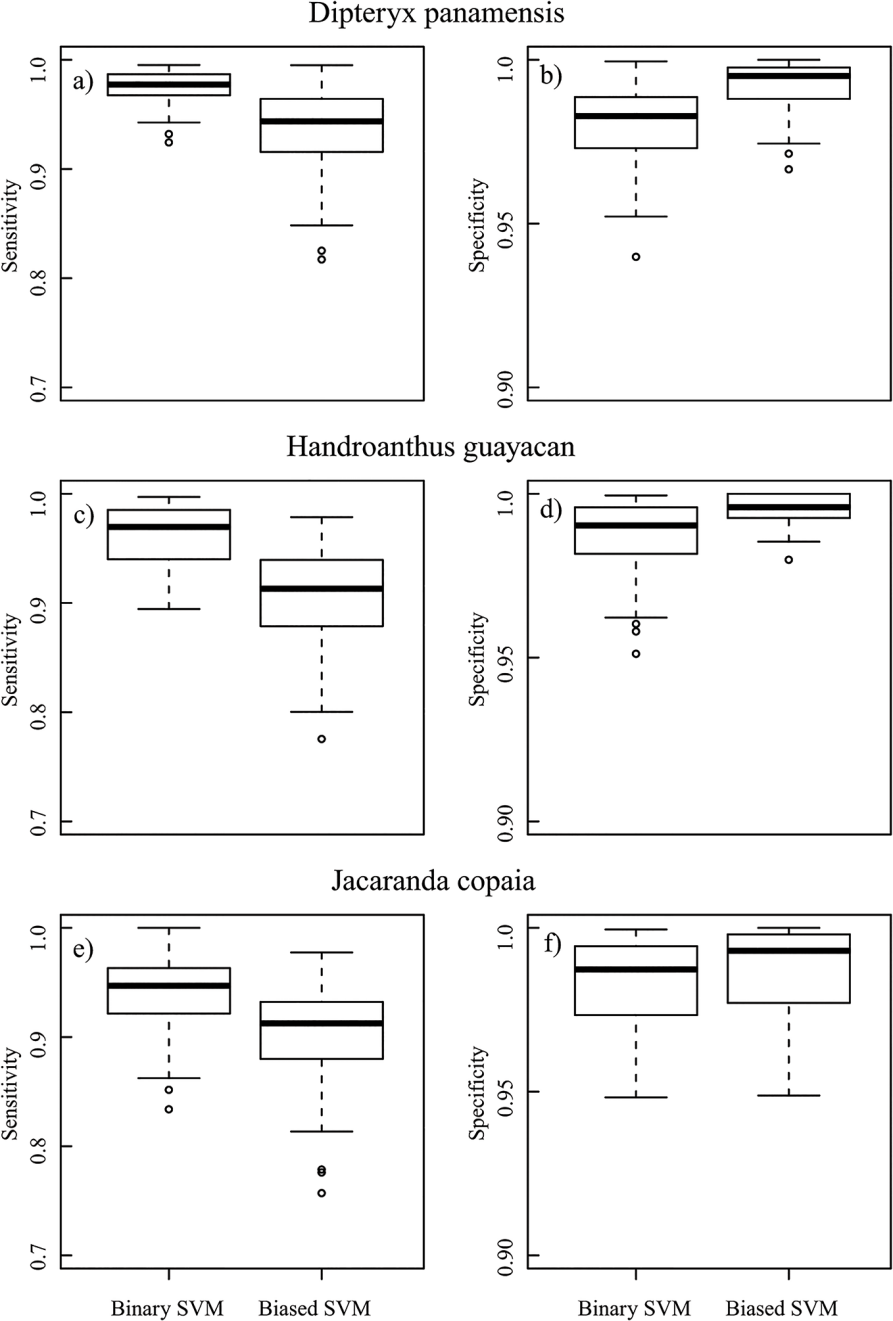
Performance of binary and biased SVM for the three focal species. Results are given for both model sensitivity (a, c, e) and specificity (b, d, f) measured on the spectra from a separate set of crowns. Sensitivity is the proportion of focal class samples that are correctly assigned to the focal class and specificity is the proportion of outlier class samples that are correctly assigned to the outlier class. Note the difference in the y-axis between sensitivity and specificity.

**Fig 4:**
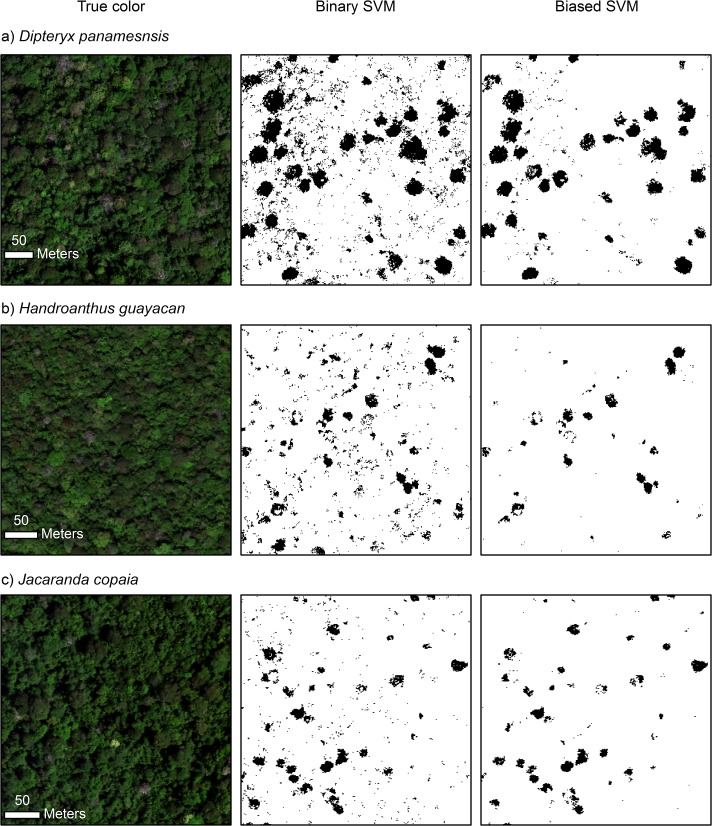
Mapped prediction results of binary and biased SVM models for the three focal species. The true-color representation of the raw imagery is on the left-hand side, results from the binary SVM models are in the center, and results from the biased SVM models are on the right-hand side. The three spectral bands used to display the true-color image are R = 640 nm, G = 550 nm, and B = 460 nm.

In general, binary SVM had higher sensitivity but lower specificity than biased SVM for the same focal species (Fig 3). The average model sensitivity for binary SVM was 0.036–0.057 higher across species than that of biased SVM. The difference was smaller for specificity, which was 0.004–0.012 higher across species for biased SVM compared to binary SVM. The maps produced from these models were consistent with this general result as more pixels were assigned to the focal class by the binary SVM models (Fig 4). However, the contrast in the amount of pixels assigned to the focal classes was quite strong, as 11.9%, 3.9%, and 4.3% of the vegetation pixels were classified as *D*. *panamensis*, *H*. *guayacan*, and *J*. *copaia*, respectively, using the binary SVM models versus only 4.1%, 0.7%, and 1.0%, respectively, using the biased SVM models. Accordingly, the binary SVM maps were noticeably noisier than those produced from biased SVM (Fig 4). We found the higher precision provided by the biased SVM models to be desirable for our mapping application; therefore, we chose biased SVM as the basis for the final species classification model.

### Focal species mapping

**Fig 5:**
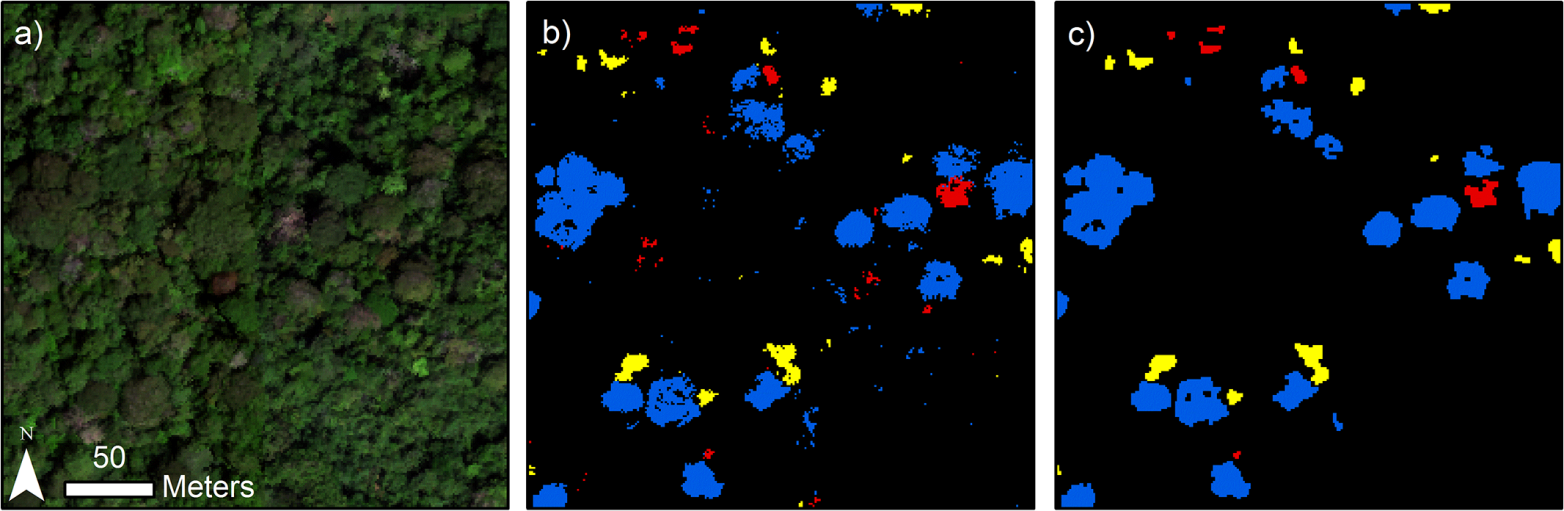
Mapped results of the multi-species model before and after applying the contextual filters. The panels are (a) true-color representation of the raw imagery, (b) prediction results for the multi-species classification model, and (c) prediction results after undergoing crown smoothing and size filtering. The three spectral bands used to display the true-color image in (a) are R = 640 nm, G = 550 nm, and B = 460 nm. The colors used to represent the species in (b) and (c) are blue = *D*. *panamensis*, red = *H*. *guayacan*, and yellow = *J*. *copaia*.

The tie-breaking three-species classification model showed good classification performance, with an overall accuracy of 97.9% based on five-fold crown-level cross-validation. However, the tie-breaking model was rarely needed to break prediction ties. When the biased SVM models were applied to the imagery, 5.6% of the vegetation pixels were assigned to at least one of the focal species classes and of these, only 2.1% were assigned to more than one focal species class. Thus, the tie-breaking model was used to predict the identity of only 0.1% of the vegetation pixels. We estimated that the final classification model, which included the three individual biased SVM models plus the tie-breaking model, had pixel-level producer’s accuracies (sensitivity for the focal class × 100) of 97.4%, 94.3%, and 93.9% for *D*. *panamensis*, *H*. *guayacan*, and *J*. *copaia*, respectively, based on five-fold crown-level cross-validation. A portion of the mapped results of the final multi-species classification is shown in Fig 5B.

**Fig 6:**
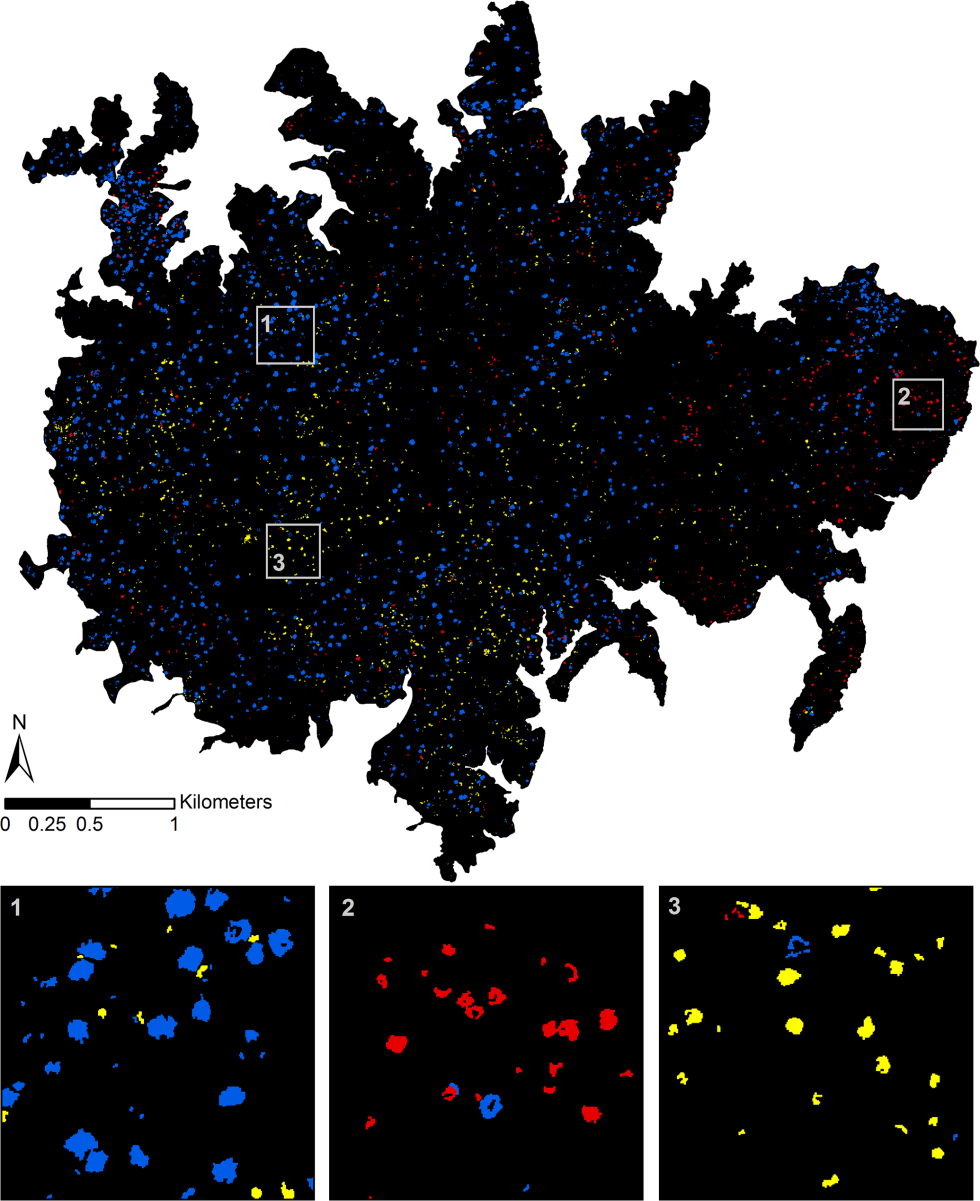
Prediction results for the three focal species across BCI. Blue = *D*. *panamensis*, red = *H*. *guayacan*, and yellow = *J*. *copaia*. Insets show close-up of results for high-density areas of each focal species.

The contextual filters reduced the number of pixels assigned to the focal species classes by 12.8%, 26.4%, and 23.1% for *D*. *panamensis*, *H*. *guayacan*, and *J*. *copaia*, respectively. The number of objects (contiguous areas) assigned to the three species was reduced much more drastically by 90.4%, 88.1%, and 85.1%, respectively (Fig 5C). This occurred because the filters removed a large quantity of small objects, often only one pixel in size. After filtering, there were a total of 2,107 predicted crown objects of *D*. *panamensis*, 837 of *H*. *guayacan*, and 1,405 of *J*. *copaia* across the entire island (Fig 6).

### Field validation

Field validation indicated that our map of predicted crown objects was very precise. We visited 39 crown objects assigned to *D*. *panamensis*, 38 of *H*. *guayacan*, and 32 of *J*. *copaia*. Of these, only one crown object assigned to *D*. *panamensis* and two assigned to *H*. *guayacan* were found to be incorrect. This resulted in an estimated false-positive error rate of 2.6% for *D*. *panamensis*, 5.3% for *H*. *guayacan*, and 0% for *J*. *copaia*.

We observed some individuals of each species in the field that were not detected by our model. Six *D*. *panamensis* crowns were encountered that were not predicted by our maps. Of these, one was found to have been cut by the NDVI filter (through inspection of the CAO imagery), one had interference by lianas, and two were small (~ 5 m in diameter). We encountered nine crowns of *H*. *guayacan* which were not predicted, of which one had low NDVI, one had interference by lianas, and one was small. We also encountered eight *J*. *copaia* crowns in the field that were not predicted, seven of which were small. Most of the small non-detected *J*. *copaia* crowns had been swept out of the prediction map by the image processing steps designed to remove noise and small objects. Both the false-positive rates and the relative lack of plausible reasons for crown omission errors indicate that *H*. *guayacan* was the most troublesome of the three focal species to detect.

## Discussion

### Model performance

Our final classification model was found to perform very well, which was especially remarkable considering that it was produced using 138 training crowns from only the species that were mapped. The crown object map that we produced from the classification results was highly reliable in the sense that it had a very low false-positive error rate for predicted crown objects. The classification model had high pixel-level producer’s accuracies for the three focal species, though some of the model’s sensitivity in detecting the focal species was certainly lost after the contextual filters were applied to the model results to create the map of crown objects. This was evidenced by the fact that omission errors were observed for crowns of each species while we were validating the maps in the field.

When observing focal species crowns in the field, it could be quite unclear as to what constituted an omission error. With examples from each of the focal species, we observed crowns in the field that were not predicted or only partially predicted by our model that were highly contaminated or partially or fully covered by lianas. Additionally, it was not uncommon that a crown object appearing to represent a single crown both in the CAO imagery and in the prediction map was found to be two or more adjacent individuals of the same species. Both of these occurrences are important from an ecological perspective because they contribute to an under-estimation of the number of individuals of a species, though we do not consider them to be errors from a remote species detection perspective. Some of the omitted crowns, such as many of those of *J*. *copaia*, were simply small, and may have been captured by our model if we had used a less conservative size filter (though this may have introduced false-positives). There were other instances from each species where crowns were missed by the prediction model for no apparent reason of interference by other species, insufficient size, or lower NDVI, which occurred most often for *H*. *guayacan*.

The three species examined here have previously been mapped across the island either by their flowering characteristics or through visual interpretation of aerial photographs. Flowering crowns of *H*. *guayacan* were mapped across BCI using two Quickbird images from different years with an automatic approach combining spectral angle mapping and one-class SVM [47]. Using visual interpretation of aerial photographs, *D*. *panamensis* crowns were identified based on canopy structure [48], and *H*. *guayacan* and *J*. *copaia* crowns were identified through their flowering traits [49,50]. Additionally, J. R. Kellner produced island-wide maps of all three focal species (the same used to obtain focal species training data used in this study) based on crown flowering using multispectral satellite imagery with an automatic detection method [36]. The island-wide distributions of the three focal species produced by our species detection model resemble those found in these previous studies [36,47,48,50]. However, our model detected many more crown objects than the number of crowns reported in these studies (Table 2). This suggests that our model, which uses imaging spectroscopy and is not limited to detecting flowering individuals, was more powerful. However, caution is warranted with these comparisons as our results pertain to crown objects and not individuals *per se* (though the same logic likely applies to other studies as well).

**Table 2:** The number of crown objects detected in the present study compared to previous studies.

*D*. *panamensis*	*H*. *guayacan*	*J*. *copaia*	Study
2107	837	1405	Present study
	706		Sanchez-Azofeifa et al. [46]
	688	977	Garzon-Lopez et al. [49]
904	804	432	J. R. Kellner [50]

The forest of Barro Colorado Island posed several challenges to remote species mapping; namely, high species diversity of the canopy, high prevalence of lianas, and a continuous canopy layer in which individual tree crowns are difficult to distinguish. We attribute the success of remote species mapping in this forest to two main factors: i) a shift from multi-species to single-species classification, and ii) performing species classification at the pixel-level, with crown objects identified after classification.

### Single-species detection

A single-species classification approach has considerable advantages over the traditional multi-species classification framework when the interest is in mapping one or a few focal species. Single-species classification can increase model performance with respect to the focal species and decrease the overall amount of data required to train the species that are not of interest (or in the case of biased SVM, it eliminates this requirement altogether) [25,26]. However, a focus on single-species detection need not restrict mapping to only one species; single-class classification models can be combined in different ways to make a multi-class classification [51]. For example, Muñoz-Marí et al. [52] combined several support vector domain description classifiers (very similar to one-class SVM) into a multi-class model to map land cover classes. One-against-all binary SVM and biased SVM were found to outperform one-class SVM for remote species identification [26], and for this analysis, we combined biased SVMs to create a multi-species identification model. Thus, improving single-species classification models can be one important route towards building more effective multi-species classification models.

Both binary and biased SVM models yielded reasonable performance with this dataset. Tested on a separate crown dataset, we found that binary SVM provided greater sensitivity but slightly lower specificity than biased SVM. However, comparing the performance of these methods in this way may give an incomplete view of their relative performances. When the models were applied to the imagery, binary SVM assigned approximately three to six times more pixels to the focal species compared to biased SVM. We may expect that small differences in model specificity have a large impact when the model is applied to the image as the non-focal species make up the majority of the landscape. However, it is also possible that noisy results from the binary SVM model could be an artifact of less-than-complete representation of the non-focal vegetation by our training dataset of over 300 crowns. This phenomenon was suggested by the results of a previous study comparing binary and biased SVM for remote species identification of five focal species in a savanna ecosystem [26]. In that study, binary SVM generally performed better than biased SVM, except that binary SVM produced noticeably noisier maps than biased SVM for one of the focal species. This was hypothesized to be caused by less representative training of the non-focal class for the higher-diversity riparian areas in which that particular focal species was found. In the current study we found that biased SVM consistently produced more precise maps than binary SVM, which supports the hypothesis that biased SVM has an advantage in high diversity landscapes.

Thus, although our preliminary experiments using cross-validation of the training dataset indicated that the binary SVM models had very high specificity, maps produced by these models were not as precise as we wished. The precision of binary SVM would likely improve if more training data were gathered for the non-focal species, but pursuing this track may be impractical in such an ecosystem. With high tree species diversity it becomes very costly to comprehensively sample the non-focal tree species, and in the BCI forest there is also a high diversity of other vegetation types, mostly lianas, that contributes to the non-focal class in the canopy. Biased SVM offers a solution to this problem by comprehensively sampling the spectra of all vegetation present and using this information to constrain the focal species class. Whereas the details of optimizing and implementing biased SVM for single-species detection were previously unclear, recent work exploring possible alternatives has made this method more straightforward to use for remote species mapping [26]. It now appears that biased SVM, through its ability to constrain the focal class independently of the quality of the outlier training data available, is not only a viable alternative but likely a preferred alternative to binary SVM when performing remote species mapping in very high diversity ecosystems.

### Delineation of tree crowns

The automatic delineation of individual tree crowns, or crown segmentation, has long been an obstacle for operational species mapping. Segmentation of individual crowns has generally been viewed as the first step before applying species classification to an image [7,53–56]. This is not without reason, as some studies have found that accuracy of species classification models was higher when classification was performed on crowns instead of pixels; for example, by using the mean spectra from crowns for classification or majority vote of the pixel class assignments [7,35]. Additionally, a map of crown identities may be more ecologically meaningful than a map of pixel identities.

Crown segmentation may rely on structural differences among crowns detected by LiDAR or indicated by shadow, spatial separation of crowns, or spectral differences among crowns. Segmentation of individual tree crowns has been successfully accomplished in many places where structural differences among crowns facilitate crown separation, such as coniferous forest, savannas, or urban areas, e.g., [20,35,53,56–58], and this is especially facilitated by the use of LiDAR. In closed-canopy, non-conifer forests where there are fewer structural clues, spectral differences have been emphasized as a way to differentiate crowns, e.g., [18,59]. Segmentation of individual tree crowns based on spectral data alone is extremely difficult, and to date there has been very limited success delineating individual tree crowns in tropical forests (though progress is being made; see [59,60]). This may partially explain why some crown-level species identification models have not made it to the mapping step despite promising classification results, e.g., [7,15].

The closed-canopy and high prevalence of lianas in the BCI forest meant that individual crowns were difficult to observe in the image. However, during the model validation field campaign we found many instances where the pixel-level species predictions correctly determined the shape and size of individual tree crowns (more specifically, the shape and size of the crown area uncovered by lianas), even where *a priori* visual interpretation had failed. Therefore, we found that it was preferable to train a pixel-level species classifier and use the prediction results to dictate the spatial coverage of the focal species crowns. This approach had the advantage that errors due to incorrect delineation of tree crowns were not fed into a species identification model. It also suited our objectives because we were only interested in delineating crowns of the focal species, rather than delineating all crowns on the island. Although we could not eliminate cases where a single crown object identified by our procedure was actually two or more adjacent conspecific individuals (as observed in the field), this limitation is unlikely to be overcome using existing methods with spectral data alone. Our mapped crown results show that automatic crown segmentation can be accomplished for selected species in a closed-canopy, high diversity tropical forest when paired with species identification as a first step, and that this strategy may be a preferable alternative to attempting to perform individual tree crown segmentation based on raw spectral data followed by object-based classification of crowns.

### Remote species mapping in the tropics

In their review of high resolution remote sensing in tropical forests, Nagendra and Rocchini [22] argued that high spatial resolution remote sensing data are best suited to accurately locating features within an image and less well suited for species identification. They concluded that high-spatial resolution data may make it more difficult to characterize the spectral signatures of different species to perform species classification, citing the problem of variability among pixels within the same crown when pixels are smaller than the crown being identified. However, we found that the high spatial and spectral resolution data are suitable for both accurate feature location and species identification, and that within-crown spectral variability does not preclude species classification of pixels. A possible explanation for this disparity could be the recent shift from endmember-based classification methods such as spectral angle mapper, which use one or a few exemplar spectra for each class, towards classification methods such as linear discriminant analysis, random forest, and SVM, which can accommodate greater within-class variability. The latter two methods are even able to accommodate complex, non-normal, multi-modal within-class variation.

Here we showed that remote species identification can be successfully achieved in a diverse, closed-canopy tropical forest using high spatial resolution imaging spectroscopy data. We produced an accurate crown object map of three focal species that was found to be very precise. The recently developed biased SVM performed well as the basis for our final multi-species classification model, and its performance was judged superior to the current standard method of binary SVM for our dataset. Thus our final multi-species classification model had the considerable advantage of only requiring representative training data from the species of interest, which can greatly ease the burden associated with producing species classification models, especially in high diversity ecosystems. Our method of producing crown objects from the pixel-level classification results also has implications for automatic tree crown delineation, indicating that individual crowns in can be delineated in high diversity, closed-canopy tropical forests when coupled with species identification. Further experimentation is needed to determine whether these methods will prove useful for remote species mapping in other ecosystems, especially those with high species diversity and a continuous closed canopy.

## Supporting Information

S1 Fig Overall performance (F-score) of binary SVM models for the three focal species as the number of training crowns was varied for both the focal species and the outlier species classes. *n*_*f*_ indicates the number of crowns of the focal species class and *n*_*o*_ indicates the number of crowns for the outlier species class.
(EPS)

S1 Methods Methods and results for the analysis of the training data amount and balance for binary SVM. (DOC)
