# Supplementary material for: Operational Tree Species Mapping in a Diverse Tropical Forest with Airborne Imaging Spectroscopy
Source: PLoS One. 2015 Jul 8;10(7):e0118403. doi: 10.1371/journal.pone.0118403 (PMC4496029; doi:10.1371/journal.pone.0118403)
Supplement: S1 Methods — (DOC) [file pone.0118403.s002.doc]

**Supplementary Materials**

*Training data amount and balance for binary SVM*

*Method* – We evaluated the performance of one-against-all binary SVM when models were built using different numbers of focal and outlier crowns. For a given focal species, all crowns belonging to that species were designated as the focal class, while all non-focal species crowns from the field data collection campaign were designated as the outlier class. The number of crowns used to train the focal class (*n_f_*) was varied among {10, 15, 20, 25, 30} and the number of crowns used to train the outlier class (*n_o_*) was varied among {50, 100, 150, 200}. For each of the 20 combinations of *n_f_* and *n_o_*, *n_f_* focal species crowns and *n_o_* outlier species crowns were randomly drawn from the crown dataset. A binary SVM was constructed using the RBF kernel, and the parameters were tuned through an exhaustive grid search over the values γ ∈ {*e*^-12^, *e*^-11^, …, *e*^-6^} and *C* ∈{*e*^5^, *e*^6^, …, *e*^15^}. Each parameter combination was judged via the F-score on the focal class, calculated by five-fold cross validation.

Model accuracy was assessed on a separate set of test crowns, which was constructed by randomly selecting five separate focal crowns and 50 separate outlier crowns. Performance measures (F-score, recall accuracy, and precision) were based on the number of pixels in the test dataset that were correctly classified. The procedure of randomly selecting training and test crowns was repeated 100 times for each of the 20 combinations of *n_f_* and *n_o_*. However, optimization was performed only once for each of the 20 combinations.

*Results* – Overall model performance, measured as the F-score on the focal class, improved with increasing numbers of focal species crowns and outlier crowns (Figure S1). Therefore, the best model performance was given by the models that were constructed from 30 focal species crowns and 200 outlier crowns. This ratio of focal to outlier crowns (0.15) that was found to perform well was also very similar to the ratios of focal to outlier crowns in the entire dataset (0.149, 0.142, and 0.132 for *D. panamensis*, *H. guayacan*, and *J. copaia*, respectively). Therefore, we concluded that the using the full crown dataset would work well for creating binary SVM models for these species, as the full dataset would provide more training data, but would also preserve a very similar balance between the focal and outlier class.
